# Nanoparticles of Chitosan Loaded Ciprofloxacin: Fabrication and Antimicrobial Activity

**DOI:** 10.15171/apb.2017.051

**Published:** 2017-09-25

**Authors:** Zahra Sobhani, Soliman Mohammadi Samani, Hashem Montaseri, Elham Khezri

**Affiliations:** ^1^Department of quality control, Faculty of pharmacy, Shiraz University of Medical Science, Shiraz, Iran.; ^2^Center for nanotechnology in drug delivery, Faculty of pharmacy, Shiraz University of Medical Science, Shiraz, Iran.; ^3^Department of pharmaceutics, Faculty of pharmacy, Shiraz University of Medical Science, Shiraz, Iran.

**Keywords:** Chitosan, Ciprofloxacin, Nanoparticle, Tripolyphosphate

## Abstract

***Purpose:*** Chitosan is a natural mucoadhesive polymer with antibacterial activity. In the present study, chitosan (CS) nanoparticles were investigated as a vehicle for delivery of antibiotic, ciprofloxacin hydrochloride.

***Methods:*** Ionotropic gelation method was used for preparation chitosan nanoparticles. The effects of various factors including concentration of CS, concentration of tripolyphosphate (TPP), and homogenization rate on the size of nanoparticles were studied. The effects of various mass ratios of CS to ciprofloxacin hydrochloride on the encapsulation efficiency of nanoparticles were assessed.

***Results:*** The particles prepared under optimal condition of 0.45% CS concentration, 0.45% TPP concentration and homogenizer rate at 6000 rpm, had 72 nm diameter. In these particles with 1:0.5 mass ratio of CS to ciprofloxacin hydrochloride, the encapsulation efficiency was 23%. The antibacterial activity of chitosan nanoparticles and ciprofloxacin-loaded nanoparticles against E.coli and S.aureus was evaluated by calculation of minimum inhibitory concentration (MIC). Results showed that MIC of ciprofloxacin loaded chitosan nanoparticles was 50% lower than that of ciprofloxacin hydrochloride alone in both of microorganism species. Nanoparticles without drug exhibited antibacterial activity at higher concentrations and MIC of them against E.coli and S.aureus was 177 and 277 µg/ml, respectively.

***Conclusion:*** Therefore chitosan nanoparticles could be applied as carrier for decreasing the dose of antibacterial agents in the infections.

## Introduction

Management of infectious disease can be improved by prolonging the contact time of antibiotics with the microorganism surface. The continuous search for potential antimicrobial agent has led to identification of antimicrobial biomaterials that are based on polymers or their composites.^1,2^ Chitosan [poly B-(1–4)-2-amino-2-deoxy-d-glucose] as a poly cationic biopolymer has high antimicrobial activity.^3,4^ This natural polysaccharide possesses useful properties such as non-toxicity, biodegradability, low price, high biocompatibility and non-antigenicity.^3-10^ The proposed mechanism for its antimicrobial action is binding to the negatively charged bacterial cell wall, with consequent destabilization of the cell envelope and altered permeability, followed by attachment to DNA with inhibition of its replication.^1,11,12^ Additionally through its positive ionic interactions with the negative charges of the cell surface membranes the drug can be exposed to microorganisms for a longer time.^11,13,14^ Furthermore, it has been shown that chitosan and its derivatives can act as antibacterial agents against both Gram-negative and Gram-positive bacteria.^14^ Regarding to these points, the potency of antibacterial agents against microorganisms may be increased by loading them into the chitosan nanoparticles. Nanoparticulate drug delivery systems may improve therapeutic efficacy through enhancing the antibiotic concentration in the microorganism without increasing the dose of administrated antibiotic.^15^

In the present work we developed ciprofloxacin-loaded chitosan nanoparticles and evaluated their physicochemical properties. After that, the antibacterial activity of selected formulation with appropriate physicochemical specifications against ciprofloxacin susceptible bacteria including *Escherchia coli* as a Gram-negative strain and* Staphylococcus aureus* as a Gram-positive strain was evaluated.

## Materials and Methods

### Materials

Low molecular weight chitosan (CS) and tripolyphosphate (TPP) was purchased from Sigma Aldrich Co. (USA_)._ Ciprofloxacin hydrochloride was obtained from Exir pharmaceutical Co. Tryptic soy broth culture was purchased from Merck Co. (Germany). *Escherchia coli* (ATCC 25922) and *Staphylococcus aureus* (ATCC 25923) were kindly donated by Doctor Alborzi Clinical Microbiological Research Center. All other reagents were of analytical grade and used as received.

### Preparation of ciprofloxacin-loaded chitosan nanoparticles

Chitosan nanoparticles were prepared using ionic gelation method. Nanoparticles were prepared by addition of TPP aqueous solution to the CS aqueous solution (in 1% v/v acetic acid) dropwise under stirring at room temperature, until faint turbidity.^16^ Orthogonal experiment was designed for evaluation of the effect of CS concentration, TPP concentration, and stirring speed on the particle size. Three factors and their three levels were shown in Table 1. The resulting experimental design consisted of 9 runs, was shown in Table 2.

Two selected formulations which had the lowest particle size were used to fabricate ciprofloxacin-loaded CS nanoparticles by the same method, except that ciprofloxacin was added into CS solution at different polymer:drug ratio (W:W) prior to the addition of TPP solution.

**Table 1:** Factors and levels of orthogonal test

Levels	Factors		
	Chitosan Concentration (%)	TPP Concentration (%)	Stirring speed (rpm)
1	0.2	0.3	6000
2	0.3	0.45	9000
3	0.45	0.675	13500

**Table 2:** Designed formulations of orthogonal experiments

Formulations	Factors		
	Chitosan Concentration (%)	TPP Concentration (%)	Stirring speed (rpm)
1	0.2	0.3	6000
2	0.2	0.45	9000
3	0.2	0.675	13500
4	0.3	0.3	9000
5	0.3	0.45	13500
6	0.3	0.675	6000
7	0.45	0.3	13500
8	0.45	0.45	6000
9	0.45	0.675	9000

### Characterizations of nanoparticles

#### Particle size 

Particle size distribution of CS nanoparticles was determined using laser diffraction particle size analyzer (Shimadzu, Model SALD-2101, Japan) at room temperature. The polydispersity index (PDI) of nanoparticles was calculated by the equation 1:

(Equation 1) $$Span=\frac{\left(D90-D10\right)}{D50}$$

Where D90, D50 and D10 designates that the particle size for which 90%, 50% and 10% of the particles are smaller than these volumes, respectively.

#### Drug encapsulation efficiency

The encapsulation efficiency was analyzed according to the procedure reported by Cevher et al. (2006).^5^ After drug loading, nanoparticles were separated from the suspension by ultracentrifugation (Hettich, Model Mikro220R, Germany) at 15500 rpm and 4°C for 30 min. The amount of free ciprofloxacin in the supernatant was measured by UV-Vis spectrophotometer (PG instruments, Model T80+, England) at 270 nm. The encapsulation efficiency (EE) was calculated by the equation 2:

(Equation 2) $$EE\%\;=\;\frac{\left(\text{T}-\text{F}\right)}{\text{T}}\times 100$$

Where F is the free amount of drug in the supernatant and T is total amount of drug added into CS solution. A blank sample was made from nanoparticles without loaded drug but treated similarly as the drug-loaded nanoparticles.

All analyses were carried out in triplicate.

#### Statistical analysis

Drug encapsulation efficiencies between different formulations were compared. To determine the optimum formulation for further studies, statistical analysis was performed using the Student’s *t*-test. Differences were considered significant at *P* <0.05.

#### Differential scanning calorimetric (DSC) analysis

Thermal analysis using a DSC method was used to characterize the thermal behavior of the chitosan, TPP, ciprofloxacin HCl, blank nanoparticles, and drug-loaded nanoparticles employing differential scanning calorimeter (TA Instruments, Model 302, Germany). Samples were accurately weighed into standard aluminum pans and sealed. All samples were run at a heating rate of 10°C/min over a temperature range of 25–300°C under nitrogen atmosphere. An empty pan, sealed in the same way as the sample, was used as a reference.

#### In vitro drug release 

A comparative in vitro drug release study was carried out in three different pH values of 5.0, 6.8 and 7.4 in phosphate buffer solution (PBS). Drug-loaded chitosan nanoparticles, suspended in 5.0 ml PBS was placed in the dialysis membrane (cutoff: 12kDa, Sigma Aldrich, USA, supplier: Kimia Teb Tajhiz, Shiraz, Iran) tied at both ends and immersed in the cell containing 100 ml of PBS. The cell was put into shaker incubator (Farazma, Iran) under the condition of 37°C and 25 rpm. To determine the concentration of drug in the receiving compartment, samples (5ml) were withdrawn from the cell at scheduled time points and replaced by the same volume of fresh pre-warmed PBS solution to avoid saturation phenomena and maintain the sink condition. Samples were analyzed at 270 nm. The release dosage at each moment was calculated and drawn into cumulative release curve.

#### Assays for antibacterial activity

#### Determination of minimum inhibitory concentration (MIC)

All glassware used for the tests were sterilized in an autoclave at 121°C for 15 min prior to use. All particles were sterilized by exposing to UV radiation for 60 min prior to the tests.^17^

The minimum inhibitory concentration (MIC) of ciprofloxacin and ciprofloxacin-loaded CS nanoparticles were determined by a turbidometric method using Tryptic soy broth (TSB), against *E.coli* and *S.aureus* strains. Drug concentrations ranging from 31.25-4000 ng/ml for *S.aureus* and 5-640 ng/ml for *E.coli* was used. All cultures were inoculated with final bacterial concentration of 10^5^ CFU/ml. After incubating for 24 hours at 37°C, samples were evaluated. The lowest concentration that inhibited the growth of bacteria was considered as the MIC.

To determine the MIC of chitosan nanoparticles without any drugs against *E.coli* and *S.aureus*, different particle concentrations were prepared and aseptically inoculated and incubated for 24 hours at 37°C.

## Results

### Particle size

The mean size and polydispersity index (PDI) of CS nanoparticles in aqueous medium measured by dynamic laser light scattering showed in Table 3.

Formulations No.4 and No.8 that had lower particle sizes were selected for further studies.

**Table 3:** Particle size and polydispersity index of CS nanoparticles

No.	Factors			Experimental result	
	CS Conc.(%)	TPP Conc.(%)	Stirring speed(rpm)	Particle size (nm)(mean±SD)	Polydispersity index(mean±SD)
1	0.2	0.3	6000	5.77±117.33	0.208±1.290
2	0.2	0.45	9000	2.64±112	0.056±1.110
3	0.2	0.675	13500	45.13±160	0.225±1.49
4	0.3	0.3	9000	15.00±94	0.016±0.785
5	0.3	0.45	13500	92.66±623.33	0.027±0.710
6	0.3	0.675	6000	2.89±111.67	0.058±1.103
7	0.45	0.3	13500	29.51±338	0.059±0.717
8	0.45	0.45	6000	13.32±71.67	0.057±0.705
9	0.45	0.675	9000	83.74±159.33	0.169±1.001

**Abbreviations:** CS Conc., chitosan concentration; TPP Conc. tripolyphosphate concentration. Data are presented as means ± standard deviations (n = 3).

### Drug encapsulation efficiency

**Figure 1:**
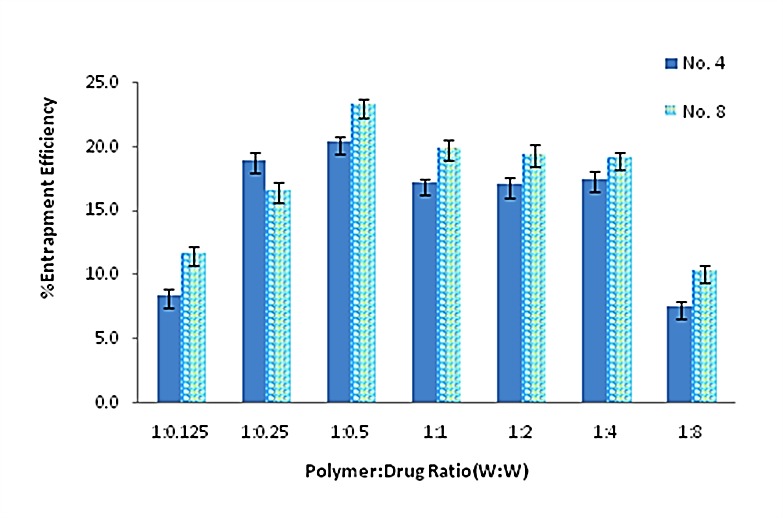
Average percent of drug entrapment efficiency of different nanoparticulate formulations. Data are presented as means ± standard deviations (n = 3). (Formulation No. 4 has 0.3% chitosan and 0.3% tripolyphosphate, and the stirring speed was 9000rpm. Formulation No 8, has 0.45% chitosan and 0.45% tripolyphosphate, and the stirring speed was 6000rpm.)

In this section, different polymer:drug ratio (W:W) ranged from 1:0.125 to 1:8 was considered as a variable factor for two selected formulations. The average percent of drug entrapment efficiency of different nanoparticulate formulations was shown in Figure 1.

The drug encapsulation efficiency in formulation No. 8 was greater than other formulations, significantly (p<0.05). This formulation with 1:0.5 polymer:drug ratio (W:W) was selected for further analysis.

### Differential scanning calorimetric (DSC) analysis

**Figure 2:**
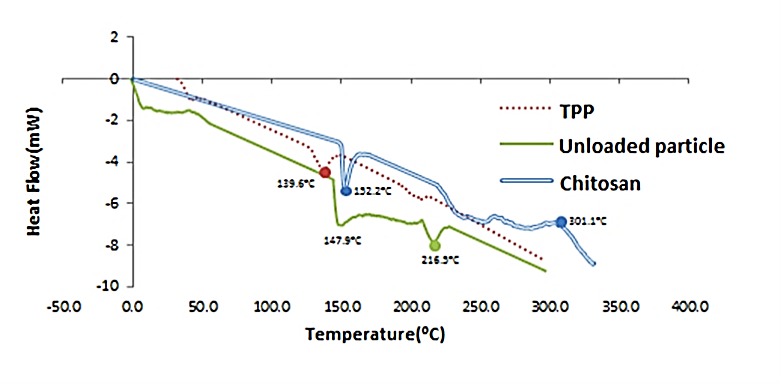
DSC thermogram of Chitosan, TPP (tripolyphosphate) and Unloaded particles.

**Figure 3:**
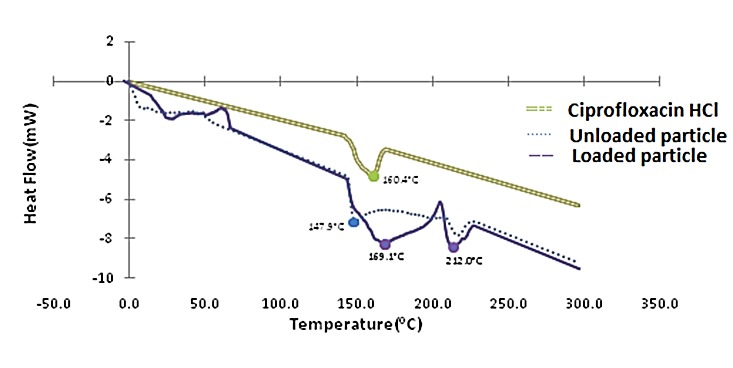
DSC thermogram of ciprofloxacin HCl, unloaded and loaded nanoparticles.

Ionic gelation between CS and TPP for formation of nanoparticles (Figure 2) and establishing the presence of ciprofloxacin in the particles was analyzed through DSC (Figure 3). In the CS thermogram (Figure 2), a sharp endothermic peak at 152.2°C and an exothermic peak at 301.1°C were in accordance with the literature‏.^3^ TPP showed endothermic peak at 139.6°C; while nanoparicles without any drugs (unloaded particles) had two endothermic peaks at 147.9°C and 216.5°C.

The physical state of the ciprofloxacin HCl inside the nanoparticles was also assessed by thermal analysis. According to the thermograms (Figure 3), ciprofloxacin HCl presented a broad endotherm centered at 160.4°C. In the DSC curves of drug-loaded nanoparticles, characteristic peaks of CS nanoparticles and ciprofloxacin HCl were joint together and seen.

### In vitro drug release studies

**Figure 4:**
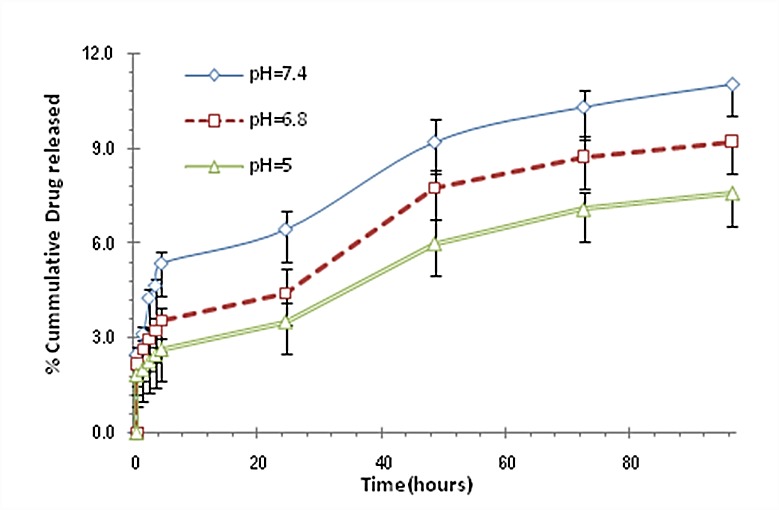
Ciprofloxacin HCl release profile from chitosan nanoparticles at three different pH values. Data are presented as means ± standard deviations (n = 3).

The release profile of ciprofloxacin HCl from ciprofloxacin-loaded CS nanoparticles at three different pH values were displayed in Figure 4. Released drug from CS nanoparticles was little and through 96 hours less than 12% of drug could be released. By decreasing the pH, the amount of released drug was decreased. The release of free drug through dialysis membrane was also evaluated and 100% of drug was permeated after 1 hour (data was not shown).

### Determination of minimum inhibitory concentration (MIC)

The antibacterial activity of ciprofloxin HCl was compared with that of ciprofloxacin HCl loaded in CS nanoparticles against Gram-positive and Gram-negative bacteria. In both microorganism strains, the MIC value were decreased significantly (Student’s *t*-test, P<0.05) by 50% when charged with ciprofloxacin HCl-loaded CS nanoparticles (80 ng/ml versus 40 ng/ml for *E.coli* and 500 ng/ml versus 250 ng/ml for *S.aureus*). The MIC values of CS nanoparticles without any drug against *E.coli* and *S.aureus* were approximately 177 and 277 µg/ml, respectively.

## Discussion

Chitosan is a natural mucoadhesive polymer with antibacterial activity. We prepared nanoparticles of chitosan by ionic gelation method containing ciprofloxacin to evaluate the possible changes in the potency of antibacterial agent. Preparation of ciprofloxacin loaded nanoparticles was performed in some studies, but the antibacterial activity of chitosan loaded ciprofloxacin was not reported elsewhere.^16,18-21^ Various concentrations of CS and TPP were examined to get the lowest particle size. The particles prepared under optimal condition of 0.45% CS concentration, 0.45% TPP concentration and homogenizer rate at 6000 rpm had 72 nm diameter. In these particles with 1:0.5 mass ratio of CS to ciprofloxacin hydrochloride, the encapsulation efficiency was 23%. The average drug entrapment efficiency of the nanoparticulate formulations was found to be increased slightly with increasing the amount of drug in polymer:drug ratios (W:W) from 1:0.125 to 1:0.5 (Figure 1). It may be due to the more drugs available which reacted with polymer. In addition, formulation No.8 had more entrapment efficiency in comparison with formulation No.4. Regarding this, No.8 included more polymer concentration (0.45% versus 0.3%) to be reacted. By increasing the amount of drug in the polymer:drug ratio from 1:0.5 to 1:8, the average entrapment efficiency of the nanoparticles was decreased. It may be due to the saturation capacity of nanoparticles. The entrapment efficiency was not high enough in different formulations. The positive charge of both CS and ciprofloxacin HCl, and partial repulsion between them, may cause these observations. In the CS thermogram (Figure 2) a sharp endothermic peak at 152.2°C and an exothermic peak at 301.1°C were related to the melting of polymer and its decomposition, respectively. In analysis between CS, TPP and unloaded particles through DSC, as expected, after the formation of ionic complex between CS and TPP, the different phenomenon from parent materials against heat was received. Endothermic centered peak of ciprofloxacin HCl was also due to the drug melting point.

Molecular weight of chitosan and crosslinking degree in nanoparticles could affect the little released drug from CS nanoparticles (Figure 4). Decrement of released drug through decreasing pH was may be due to the greater ionic interaction of CS with TPP and increasing the crosslinking density of the polymer.

Enhancing the efficacy of antibacterial agents loaded into the polymeric nanoparticles is reported in the numerous studies. These findings are related to many factors, including: facilitated penetration of drug into the bacterial cells, better delivery of the drug to its site of action, and the higher stability of the encapsulated drug into the nanoparticles.^15,18,22^ In this study the potency of ciprofloxacin HCl loaded into the CS nanoparticles against *E.coli* as a Gram-negative strain and *S.aureus* as a Gram-positive strain was increased by 50%. Usually Gram-negative bacteria are more resistant to antibiotics than Gram-positive bacteria because of the presence of especially cell wall in their structure. This certain structures limit the penetration of antibiotics to the bacterial cell.^23^ Therefore, enhancing the potency of ciprofloxacin HCl especially against *E.coli* as a Gram-negative strain by using CS nanoparticles could be a promising result for clinical studies. Physicochemical properties of ciprofloxacin-loaded chitosan nanoparticles have important effects on the antibacterial activity of the loaded ciprofloxacin. Jeong et al reported that *in vitro* antibacterial activity of ciprofloxacin-encapsulated PLGA nanoparticles against *E.Coli* is relatively lower than free ciprofloxacin.^20^ In our work, decreasing the MIC levels in both bacteria may be due to the increasing the penetration of drug by nanoparticles into the bacterial cell that inhibited the bacterial growth. Increasing the antibacterial activity of ciprofloxacin loaded in CS nanoparticles, could not be due to the antimicrobial effect of CS nanoparticles alone, because the CS nanoparticles have inhibitory effect at high concentrations. The MIC value of CS nanoparticles without any drug against Gram-negative strain was lower. The negative charge on the cell wall of the tested Gram-negative bacteria was higher than that on the tested Gram-positive bacteria, leading to more CS adsorbed and higher inhibitory effect against the Gram-negative bacteria.^4^ Chung et al reported that chitosan has a stronger effect on the Gram-negative *E. coli* than on the Gram-positive *S. aureus* in terms of the leakage of enzymes.^24^

## Conclusion

In summary, ciprofloxacin HCl-loaded chitosan nanoparticles have been prepared and characterized in the present study. The nanoparticles obtained in the present study had small particle size, which may increase the drug penetration into the bacterial cell and improve its antibacterial activity. The results showed that ciprofloxacin HCl-loaded chitosan nanoparticles could inhibit the growth of two strains of Gram-positive and Gram-negative microorganisms markedly. Their MIC values were 50% lower than MIC of free drug itself. It was anticipated that chitosan nanoparticles could be applied broadly as a carrier for antimicrobial agents in medicine for their biocompatibilities and also their antibacterial activity.

## Acknowledgments

The authors acknowledge Doctor Alborzi Clinical Microbiological Research Center for providing strains of bacteria.

## Ethical Issues

Not applicable.

## Conflict of Interest

Authors state that there is no conflict of interest. Financial support of Shiraz university of Medical Sciences is appreciated.

## References

[1] Das S, Das MP, Das J (2013). Fabrication of porous chitosan/silver nanocomposite film and its bactericidal efficacy against multi-drug resistant (MDR) clinical isolates. J Pharm Res.

[2] Sanpui P, Murugadoss A, Prasad PV, Ghosh SS, Chattopadhyay A (2008). The antibacterial properties of a novel chitosan-ag-nanoparticle composite. Int J Food Microbiol.

[3] Gomez-Burgaz M, Torrado G, Torrado S (2009). Characterization and superficial transformations on mini-matrices made of interpolymer complexes of chitosan and carboxymethylcellulose during in vitro clarithromycin release. Eur J Pharm Biopharm.

[4] Kong M, Chen XG, Xing K, Park HJ (2010). Antimicrobial properties of chitosan and mode of action: A state of the art review. Int J Food Microbiol.

[5] Cevher E, Orhan Z, Mulazimoglu L, Sensoy D, Alper M, Yildiz A (2006). Characterization of biodegradable chitosan microspheres containing vancomycin and treatment of experimental osteomyelitis caused by methicillin-resistant staphylococcus aureus with prepared microspheres. Int J Pharm.

[6] Krishna Rao KSV, Ramasubba Reddy P, Lee YI, Kim C (2012). Synthesis and characterization of chitosan–PEG–Ag nanocomposites for antimicrobial application. Carbohydr Polym.

[7] Li LH, Deng JC, Deng HR, Liu ZL, Li XL (2010). Preparation, characterization and antimicrobial activities of chitosan/Ag/ZnO blend films. Chem Eng J.

[8] Liu TY, Chen SY, Li JH, Liu DM (2006). Study on drug release behaviour of CDHA/chitosan nanocomposites-effect of CDHA nanoparticles. J control release.

[9] Tiyaboonchai W, Limpeanchob N (2007). Formulation and characterization of amphotericin b-chitosan-dextran sulfate nanoparticles. Int J Pharm.

[10] Wei D, Sun W, Qian W, Ye Y, Ma X (2009). The synthesis of chitosan-based silver nanoparticles and their antibacterial activity. Carbohydr Res.

[11] Anitha A, Deepagan VG, Divya Rani VV, Menon D, Nair SV, Jayakumar R (2011). Preparation, characterization, in vitro drug release and biological studies of curcumin loaded dextran sulphate–chitosan nanoparticles. Carbohydr Polym.

[12] Xing K, Chen XG, Kong M, Liu CS, Cha DS, Park HJ (2009). Effect of oleoyl-chitosan nanoparticles as a novel antibacterial dispersion system on viability, membrane permeability and cell morphology of Escherichia coli and Staphylococcus aureus. Carbohydr Polym.

[13] Chakraborty SP, Sahu SK, Pramanik P, Roy S (2012). In vitro antimicrobial activity of nanoconjugated vancomycin against drug resistant staphylococcus aureus. Int J Pharm.

[14] Sadeghi AM, Dorkoosh FA, Avadi MR, Saadat P, Rafiee-Tehrani M, Junginger HE (2008). Preparation, characterization and antibacterial activities of chitosan, N-trimethyl chitosan (TMC) and N-diethylmethyl chitosan (DEMC) nanoparticles loaded with insulin using both the ionotropic gelation and polyelectrolyte complexation methods. Int J Pharm.

[15] Azhdarzadeh M, Lotfipour F, Zakeri-Milani P, Mohammadi G, Valizadeh H (2012). Anti-bacterial performance of azithromycin nanoparticles as colloidal drug delivery system against different gram-negative and gram-positive bacteria. Adv Pharm Bull.

[16] Jain D, Banerjee R (2008). Comparison of ciprofloxacin hydrochloride-loaded protein, lipid, and chitosan nanoparticles for drug delivery. J Biomed Mater Res B Appl Biomater.

[17] Wiarachai O, Thongchul N, Kiatkamjornwong S, Hoven VP (2012). Surface-quaternized chitosan particles as an alternative and effective organic antibacterial material. Colloids Surf B Biointerfaces.

[18] Fawaz F, Bonini F, Maugein J, Lagueny AM (1998). Ciprofloxacin-loaded polyisobutylcyanoacrylate nanoparticles: Pharmacokinetics and in vitro antimicrobial activity. Int J Pharm.

[19] Fawaz F, Guyot M, Lagueny AM, Devissaguet JP (1997). Ciproflexacin-loaded polyisobutylcyanoacrylate nanoparticles: Preparation and characterization. Int J Pharm.

[20] Jeong YI, Na HS, Seo DH, Kim DG, Lee HC, Jang MK (2008). Ciprofloxacin-encapsulated poly(dl-lactide-co-glycolide) nanoparticles and its antibacterial activity. Int J Pharm.

[21] Page-Clisson ME, Pinto-Alphandary H, Ourevitch M, Andremont A, Couvreur P (1998). Development of ciprofloxacin-loaded nanoparticles: Physicochemical study of the drug carrier. J Control Release.

[22] Huh AJ, Kwon YJ (2011). "Nanoantibiotics": A new paradigm for treating infectious diseases using nanomaterials in the antibiotics resistant era. J Control Release.

[23] Lotfipour F, Nazemiyeh H, Fathi-Azad F, Garaei N, Arami S, Talat S (2008). Evaluation of antibacterial activities of some medicinal plants from north-west iran. Iran J Basic Med Sci.

[24] Chung YC, Chen CY (2008). Antibacterial characteristics and activity of acid-soluble chitosan. Bioresour Technol.

